# Transfusion-Transmitted Infections and associated risk factors at the Northern Zone Blood Transfusion Center in Tanzania: A study of blood donors between 2017 and 2019

**DOI:** 10.1371/journal.pone.0249061

**Published:** 2021-03-24

**Authors:** Alex Mremi, James J. Yahaya, Mramba Nyindo, Edson Mollel

**Affiliations:** 1 Department of Pathology, Kilimanjaro Christian Medical Centre (KCMC), Moshi, Tanzania; 2 Faculty of Medicine, Kilimanjaro Christian Medical University College (KCMUCO), Moshi, Tanzania; 3 Department of Histopathology and Morbid Anatomy, School of Medicine and Dentistry, The University of Dodoma, Dodoma, Tanzania; 4 Northern Zone Blood Transfusion Center (NZBTC), Moshi, Tanzania; FDA, UNITED STATES

## Abstract

**Background:**

Blood transfusion saves many people every year that would otherwise have died. The present study aimed to provide an update and insightful information regarding prevalence of the common Transfusion-Transmitted Infections (TTIs) and associated factors among blood donors in Tanzania.

**Methods:**

This was a cross-sectional study involving retrospectively collected data of blood donors from the Tanzania Northern Zone Blood Transfusion Center between 2017 and 2019. Descriptive statistics were performed to describe characteristics of the blood donors. Univariable and multivariable logistic regression analyses were performed to determine association between prevalence of TTIs and socio-demographic factors. P-value <0.05 was considered statistically significant.

**Results:**

A total of 101, 616 blood donors were included in the present study of which 85,053(83.7%) were males while 16,563 (16.3%) were females. Of all participants, the majority 45,400 (44.7%) were aged between 18 and 25 years; 79,582 (78.3%) were voluntary non-remunerated donors while 22,034 (21.7%) were replacement donors. The vast majority of them 99,626 (98%) were first time blood donors while 1990 (2%) were multiple donors. The overall prevalence of TTIs was 10.1% (10,226 out of 101,616) of which the leading was HBV accounting for 5.1% (5,264 out of 101,616). Being a replacement donor was associated with all the four types of TTIs: HIV (AOR = 1.22, 95% CI = 1.10–1.35), HBV (AOR = 1.35, 95% CI = 1.27–1.44), HCV (AOR = 1.28, 95% CI = 1.12–1.46), and syphilis (AOR = 1.33, 95% CI = 1.20–1.48).

**Conclusions:**

Our study has demonstrated that Tanzania has relatively high prevalence of TTIs compared to some countries in Sub-Saharan Africa. HBV infection seems to be the most common infection among blood donors and replacement blood donors are at a higher risk of harboring the commonest TTIs among blood donors.

## Introduction

Blood transfusion saves many people every year that would otherwise have died because of various natural diseases and disasters [1]. But unsafe blood transfusion has the potential to transmit a diverse of infections to blood recipients. These infections could be due to viruses, bacteria, protozoans, and/or prions [1]. Viral agents include human immunodeficiency virus (HIV) [1, 2], Hepatitis B virus (HBV) and Hepatitis C virus (HCV) [3]. Bacterial pathogens include among others, Yersinia, Pseudomonas, Klebsiella and Staphylococcus [4]. Plasmodium species and Babesia microti are examples of protozoan infections that can be transmitted through blood transfusion [5, 6].

A report by WHO Global Database on Blood Safety indicates that before year 2000 more than 40% of all blood donated in developing countries were not screened for TTIs, and that 80% of world population could access just 20% of the safe blood [7]. As of the year 2016, this has changed a lot with most of the African countries testing the four key diseases by 100% (i.e 44/46 test for HIV by 100%, 42/46 test by 100% for HBV, 41/46 test for HCV by 100%, 40/44 test for syphilis by 100%), this means more people have access to safe blood supply [8]. Studies done in Africa have shown that the risk of TTIs is higher compared to other settings [9]. A study done in Tanzania revealed sero-prevalence for TTIs to be 15.9% which was lower compared to a previous and similar study but among HIV sero-negative blood donors [10]

Several risk factors have been associated with the prevalence of these infections among blood donors such as implementation of donor questionnaire [11], history of high-risk behavior, history of jaundice [12], being married, low education, informal occupation, multiple sexual activity, history of blood transfusion, being male, [9]. There could be additional factors that are different in Tanzanian context.

In Tanzania, the National Blood Transfusion Services (NBTS) program coordinates and safeguards blood transfusion services since 2004. The national program currently focuses on implementing automation techniques, centralizing database and increasing regular blood donation so as to improve safety of blood supply. It also involves in step-wise accreditation of its six zones as per the international standards, so as to improve comprehensive quality management. It has six zones scattered throughout the country. Northern Zone Blood Transfusion Center (NZBTC) is one of them and it encompasses all regions on the Northern region of the Tanzania. According to 2016 NBTS data this zone had 80% of all blood donors as voluntary non-remunerated blood donors, the TTIs proportions were 1.8% for HIV, 3.4% for HBV, 1.3% for HCV and 1.5% for syphilis, respectively [13].

Furthermore, the study conducted in Northern Tanzania showed the prevalence of donor deferral among clients who donated blood was 13%. Among deferral, infection contributed more than 60%, thus it is crucial to have the up-to-date data on the prevalence of TTIs which will inform us on the deferral due to infection and enable to planning blood safety [14].

There is limited data on magnitude and risk factors for Transfusion Transmissible Infections in many developing countries including Tanzania despite the recommended efforts to screen all donated blood for infections such as HIV, HBV, HCV and syphilis. For the appropriate implementation of blood donation programmes in Tanzania, there is a need to know the prevalence of TTIs and the associated risk factors. Therefore, the present study was conceived to provide an update and insightful information regarding prevalence of the common TTIs and associated risk factors among blood donors in Northern Tanzania.

## Materials and methods

### Study design, area and period

This was a cross-sectional study involving retrospectively collected data of blood donors from the Tanzania northern zone center for blood donation between 2017 and 2019. The study duration was five months which started from January to May 2020. The study was conducted at the Northern Zone Blood Transfusion Center (NZBTC) which is located in Moshi municipal, in Kilimanjaro region in Northern Tanzania. The center was established in 2005 and among the activities done by the center includes provision of general health information and continuous education to the public on the importance of blood donation, recruiting blood donors around the zone, receiving and banking collected blood from the neighboring regions, screening the collected blood for the TTIs and distribution of blood to all health facilities found within the zone. NZBTC is one of the six national blood donation centers. The center also coordinates blood transfusion services in other three neighboring regions in the Northern zone of the country which are Tanga, Manyara and Arusha. According to the national population census of 2011/2012 report, the Tanzania Northern zone had an estimated population of about 8.6 millions [15].

### Study population, eligibility criteria and data collection

**Fig 1:**
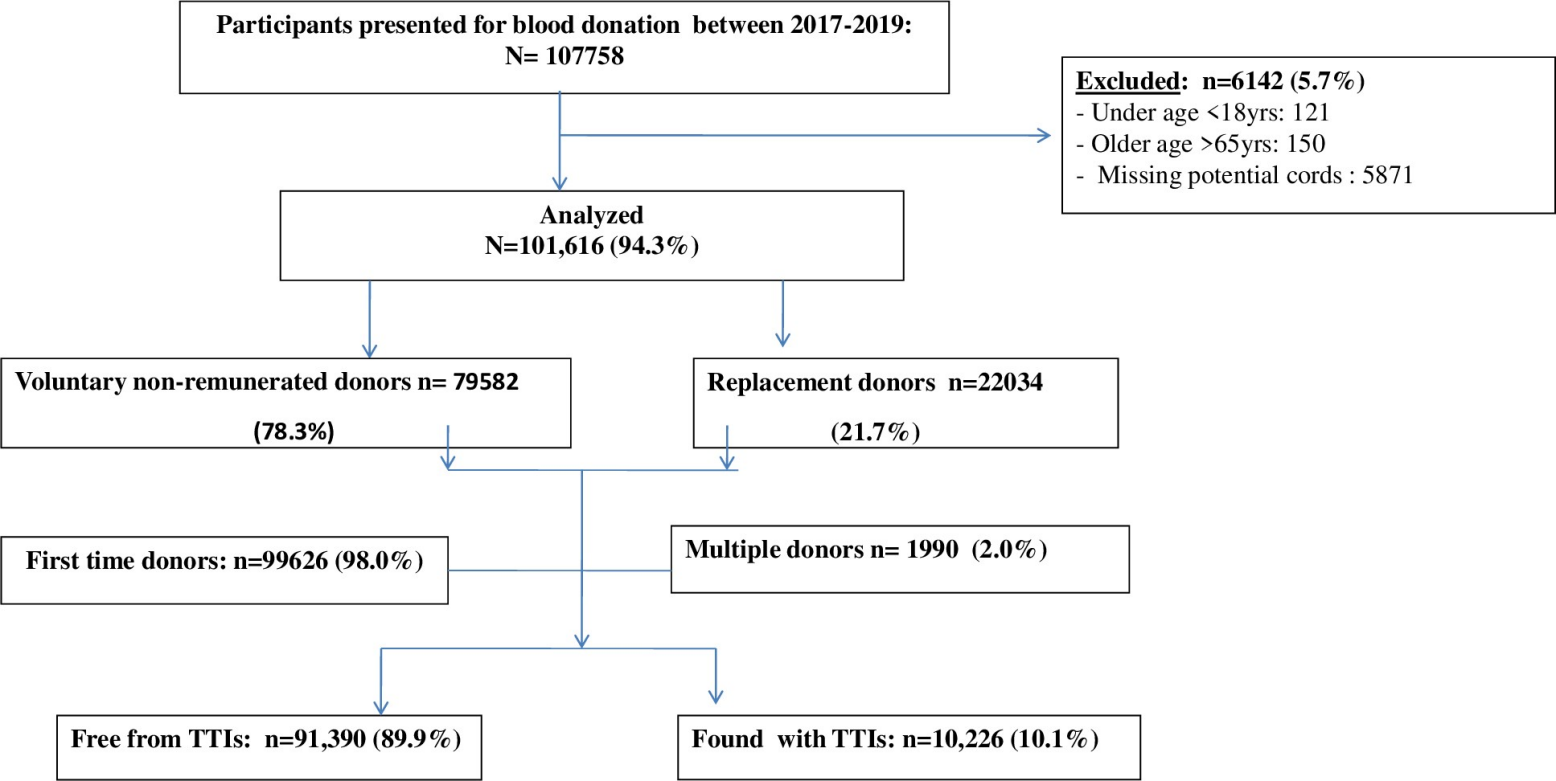
Schematic presentation of the study participants.

Socio-demographic and general health information of the blood donors registered at the NZBTC between 2017 and 2019 were extracted from the center’s database by the last author who is a physician working with the NZBTC. The quality control of the data extraction was ensured by an experienced biostatistician working with the NZBTC. The blood donation standard procedures at the NZBTC have previously been described [14]. Briefly, the potential donor candidates presenting for blood donation at the center are registered and then counseled by the trained donor counselors. Usually, the counseling process runs parallel with an assessment of the eligibility criteria for blood donation using a standardized donor questionnaire. The questionnaire inquired about the socio-demographic characteristics of participants such as age, marital status, occupation and address; donation history, the type of donor–either voluntary or replacement; and general health check of the donor in terms of diseases and risks for acquiring transmissible infections such as HIV, HBV, among many others. Included in the study were subjects aged from 18 to 65 years with body weight of not less than 50 kg; not having history of anaemia, or TTIs; and not donated blood at least 3 months for males and 4 months for females. The subjects excluded from this study included clients who did not fulfill the stipulated eligibility criteria for donation as well as those with potential missing data. Fig 1 presents the overview of the study flow chart. In this study, replacement donor referred to a friend or a family member of the recipient who donated blood to replace the stored blood used in transfusion, ensuring a consistent supply. Voluntary non-remunerated blood donor was defined as a person who gives blood on his or her own free will and receives no payment, either in the form of cash or in kind [14].

After counseling, prospective blood donors received measurements for weight, height, blood pressure and hemoglobin estimation. NZBTC facility always performs testing on each blood samples for HIV, Hepatitis B, Hepatitis C and Syphilis. The tests were done using Abbott ARCHITECT system. A previous study reported that the system has a reliable test performance with a specificity of 100% for the serological and molecular biological assays with analytical sensitivity of HBsAg, anti-HBc, anti-HBs, anti-HCV, HIV-1-p24-antigen/anti-HIV 1/2, HBV DNA, and HCV RNA detections in DBS at 98.6%, 97.1%, 97.5%, 97.8%, 100%, 93%, and 100%, respectively, [16]. Blood donation was done after the client has signed a written informed consent. Candidates with negative test results were recorded as negative and could be recalled for next donation. The blood that was found with positive or grey zone test results for the initial screening test was discarded and subjected to a duplicate repeat testing. The donors with the blood units testing positive after this duplicate repeat were deferred permanently. Subjects found with medical conditions including TTIs were promptly advised to seek an immediate medical attention.

### Statistical analysis

The collected data were exported from the excel sheet into the SPSS version 20 (IBM Statistics, Chicago-USA) and errors and missing data were managed by running cross-tabs and frequency tables. Age of the participants was presented in mean ± standard deviation and all categorical variables (sex, category of blood donor, status of sero-positivity for HIV, HBV, HCV and syphilis) were summarized in proportions. Association of the background characteristics with donors’ characteristics was performed using logistic regression analysis. Binary logistic regression was applied to determine the associated risk factors with TTIs among blood donors included in the study. Adjusted odds ratio (AOR) at 95% confidence interval (CI) was regarded to be the probability of measure of the risk for the blood donors to be found with any of the four TTIs after adjusting for the confounders in the multivariate analysis. A two-tailed P<0.05 was considered significant.

### Ethical approval

Ethical approval to carry out this study was obtained from the College Research Ethical Review Committee (CRERC) of the Kilimanjaro Christian Medical University College (KCMUCo) (Ethical approval number 2451). The study strictly abode to the international regulations for research involving human subjects as stipulated in the Helsinki declaration. The collected data were de-identified and thus anonymity of the study subjects was upheld to its maximum. Every measure for confidentiality regarding handling of the data as well as dissemination of the results was strictly adhered.

## Results

### Background information of the blood donors in the study

A total of 107,758 persons donated blood to the NZBTC between January 2017 and December 2019. Of these, 101,616 met inclusion criteria and thus were included in this study. The number of blood units donated during study period was 107, 586. The vast majority of the participants 98% (n = 99,626) were first time donors and voluntary non-remunerated donors’ category accounting for 78.3% (n = 79,582). Fig 1 presents a schematic presentation of the study participants.

Majority of the blood donors 83.7% (n = 85, 080) were males while the rest 16.3% (n = 16, 536) were females. Most of the participants 44.7% (n = 45, 400) were of age 18–25 years followed by 26.8% (n = 27, 263) who were in the age ranging between 26 and 35 years. Regarding place of residence of the blood donors included in the present study, we observed that a large proportion 32.9% (n = 33, 386) were residing in Kilimanjaro region followed by 26.2% (n = 26, 585) donors who were residents of Arusha region and the least donors 0.6% (n = 627) were coming from other regions. Moreover, the vast majority of the blood donors 78.3% (n = 79, 582) were voluntary non-remunerated donors and the remaining 21.7% (n = 22, 034) were replacement blood donors, Table 1.

**Table 1:** Socio-demographic characteristics blood donors at the Northern Zone Blood Transfusion Center, in Moshi, Tanzania (N = 101, 616).

Variables	Frequency (n)	Percentage (%)
**Year of donation**		
2017	25568	25.2
2018	37952	37.3
2019	38096	37.5
**Age (years)**		
18–25	45400	44.7
26–35	27263	26.8
36–45	18570	18.3
46–55	8543	8.4
56–65	1840	1.8
**Sex**		
Male	85080	83.7
Female	16536	16.3
**Region of residence**		
Arusha	26585	26.2
Kilimanjaro	33386	32.9
Manyara	14368	14.1
Tanga	26650	26.2
Others	627	0.6
**Type of blood donor**		
Replacement	22034	21.7
Voluntary non remunerated	79582	78.3

### Prevalence of the different Transfusion-Transmitted Infections (TTIs) among blood donors at NZBTC (n = 101,616)

**Fig 2:**
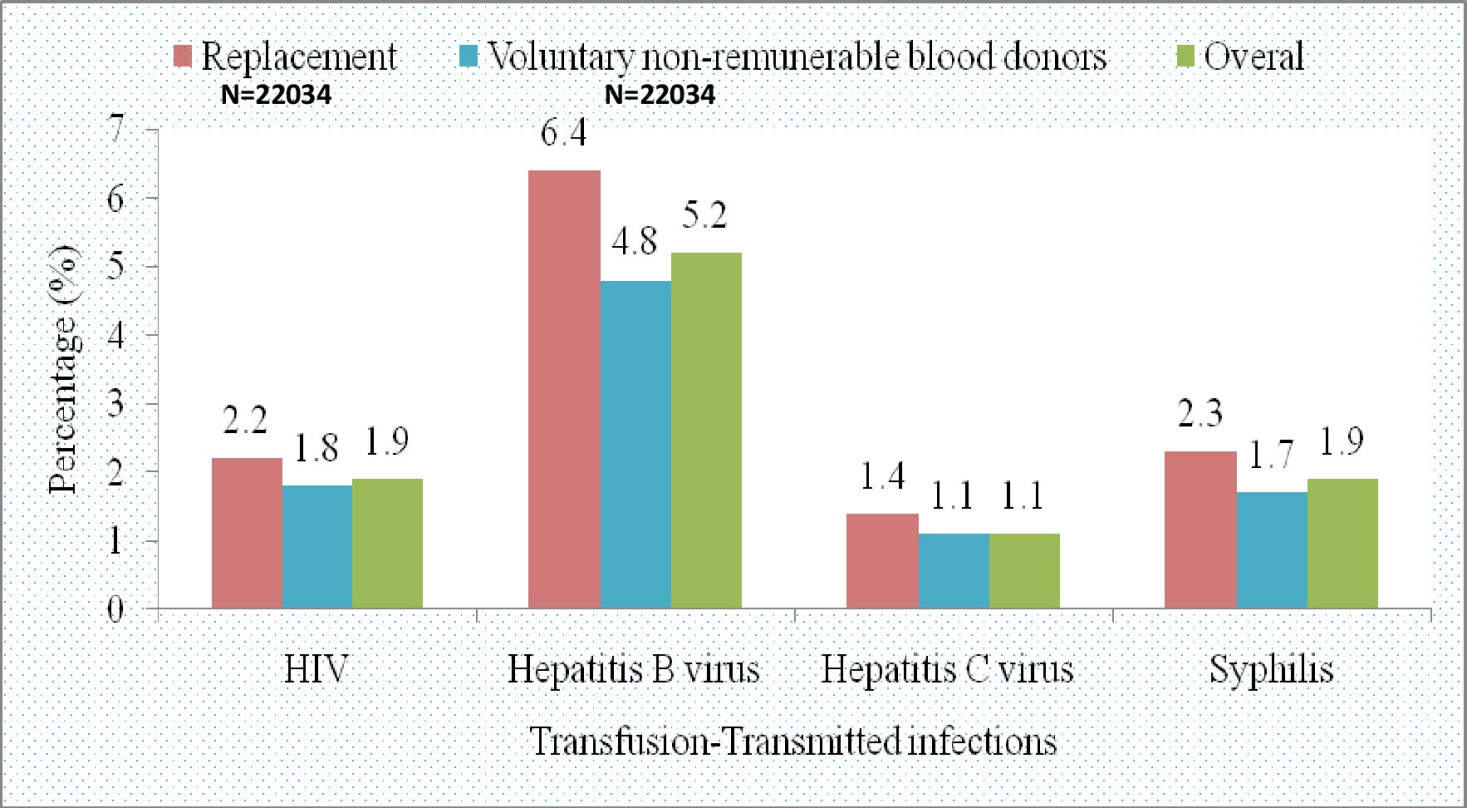
Prevalence of HIV, Hepatitis B, Hepatitis C and syphilis among replacement blood donors and voluntary non-remunerable blood donors at the NZBTC, (N = 101,616).

Of all the blood donor subjects included in the present study, a total of 10.1% (n = 10, 226) were found to harbor various types of TTIs. The prevalence of TTIs for replacement donors was relatively higher 12.3% (n = 2704) than that of voluntary non-remunerated donors 9.5% (n = 7522). Of the TTIs detected in our study, HBV was the most prevalent in both replacement and voluntary non-remunerated blood donors contributing to 6.4% (n = 1,414) and 4.8% (n = 3,850), respectively. Table 2 and Fig 2 present the different types of TTIs among the two types of blood donors reported in the present study.

**Table 2:** Association of HIV, HBV, HCV, and syphilis with age, sex, and type of blood donor (n = 10, 226).

Variables	HIV		HBV		HCV		Syphilis	
	n (%)	COR (95%CI)	n (%)	COR (95%CI)	n (%)	COR (95%CI)	n (%)	COR (95%CI)
**Sex**								
Male	1621(1.9)	1.03(0.91–1.17)	4800(5.6)	**2.07(1.88–2.28)**	992(1.2)	1.18(0.99–1.39)	1615(1.9)	**1.19(1.04–1.35))**
Female	305(1.8)	1	464(2.8)	1	164(1.6)	1	265(1.6)	1
**Age (years)**								
18–25	761(1.7)	1	1684(3.7)	1	458(1.0)	1	332(0.7)	1
26–35	531(1.9)	**1.17(1.04–1.30)**	1825(6.7)	**1.86(1.74–1.99)**	307(1.1)	1.12(0.97–1.29)	379(1.4)	**1.91(1.65–2.22)**
36–45	435(2.3)	**1.41(1.25–1.58)**	1209(6.5)	**1.81(1.68–1.95)**	250(1.3)	**1.34(1.15–1.56)**	515(2.8)	**3.87(3.37–4.45)**
46–55	173(2.0)	**1.21(1.02–1.43)**	468(5.5)	**1.50(1.35–1.67)**	107(1.3)	**1.24(1.01–1.54)**	520(6.1)	**8.80(7.64–10.14)**
56–65	26(1.4)	0.84(0.57–1.25)	78(4.2)	1.15(0.91–1.45)	34(1.8)	**1.85(1.30–2.63)**	134(7.3)	**10.66(8.66–13.13)**
**Region of residence**								
Arusha	473(1.8)	1	1159(4.4)	1	234(0.9)	1	446(1.7)	1
Kilimanjaro	588(1.8)	0.99(0.88–1.12)	1406(4.2)	0.96(0.89–1.04)	310(0.9)	1.06(0.89–1.25)	512(1.5)	0.91(0.80–1.04)
Manyara	224(1.6)	0.87(0.74–1.03)	780(5.4)	**1.26(1.15–1.38)**	152(1.1)	1.20(0.98–1.48)	356(2.5)	**1.49(1.29–1.71)**
Tanga	625(2.3)	**1.33(1.18–1.50)**	1880(7.1)	**1.67(1.54–1.80)**	453(1.7)	**1.95(1.66–2.28)**	560(2.1)	**1.26(1.11–1.43)**
Others	16(2.6)	1.45(0.87–2.39)	39(6.2)	**1.46(1.05–2.02)**	7(1.1)	1.27(0.60–2.71)	6(1.0)	**1.26(1.11–1.43)**
**Type of donor**								
Replacement	484(2.2)	**1.22(1.10–1.35)**	1414(6.4)	**1.35(1.27–1.44)**	302(1.4)	**1.28(1.12–1.46)**	504(2.3)	**1.33(1.20–1.48)**
Voluntary non remunerated	1442(1.8)	1	3850(4.8)	1	854(1.1)	1	1376(1.7)	1

HIV-Human immunodeficiency virus, HBV-Hepatitis B virus, HCV-Hepatitis C virus, COR-Crude odds ratio, CI-Confidence interval.

### Association of HIV, HBV, HCV, and syphilis with age, sex, and type of blood donor

Regarding the risk of the donors to be detected with HIV based on their age, we found that blood donors aged 26–35, 36–45, and 46–55 years were 1.17, 1.41, and 1.21 times more likely to contract HIV compared to the blood donors who had 18–25 years and the difference was significant (95% CI = 1.04–1.30, 1.25–1.58, and 1.02–1.43, respectively). Regarding region of residence with HIV infection among blood donors, we found that blood donors who were residing in Tanga region were 1.33 times more likely to be detected with HIV than any other regions and the difference was statistically signifant (95% CI = 1.18–1.50). Moreover, replacement donors were 1.22 times more likely to be detected with HIV than voluntary non-remunerated dobors with statistical difference in terms of risk (95% CI = 1.10–1.35). However, despite a slight increased risk of males to be detected with HIV (COR = 1.03) compared to females; the observed difference was not statistically signiffcant (95% CI = 0.91–1.17).

Furthermore, we found that males were 2 times more likely to be detected with HBV than females (95% CI = 1.88–2.28). Regarding the risk of the donors to be detected with HBV based on their age, we found that blood donors aged 26–35, 36–45, and 46–55 years were 1.86, 1.81, and 1.50 times more likely to contract HBV compared to the blood donors who had 18–25 years and the difference was significant (95% CI = 1.04–1.30, 1.25–1.58, and 1.02–1.43, respectively). However, despite that there was a 1.15-fold risk for males to be detected with HBV compared to females but the difference was not signiffcant (95% CI = 0.91–1.45).

Blood donors residing in Manyara and Tanga were at 1.26 and 1.67 times of being detected with HBV comparred to donors who were residing in Arusha, resectively. It was also found that donors who reside in Kilimanjaro region were protected from having HBV infection (COR = 0.96). Replacement donors were 1.35 times more likely to be detected with HBV infection than voluntary non-remunerated blood donors and the difference between the two types of blood donors was siginificant (95% CI = 1.27-.144).

The pattern of HCV infection based on region of residence and age of the blood donors was different from that of HBV infection. Only those who were residing in Tanga region were associated with HCV infection (95% CI = 1.66–2.28) and had almost 2-fold risk of being infected with HCV compare to other regions. We also found that blood donors aged 56–65 years had 1.85 odds of being infected with HCV compared to those who were aged 18–25 years and the difference was significant (95% CI = 1.85–2.63). Additionally, donors who were aged 36–45 and 46–55 years were 1.34 and 1.24 times more likely to be found with HCV than donors who were aged 18–25 years, respectively. Also replacement blood donors were 1.28 times more likely to be detected with HCV than voluntary non-remunerated blood donors and the difference between the two types of blood donors was siginificant (95% CI = 1.12–1.46).

Males were 1.19 times more likely to be detected with syphilis than females and the differnce was signficant (95% CI = 1.04–1.35). Also replacement blood donors were 1.33 times more likely to be detected with HCV than voluntary non-remunerated blood donors and the difference between the two types of blood donors was siginificant (95% CI = 1.2–1.48). moreover, the age groups 26–35, 36–45, 46–55, and 56–65 were associated with detection of syphilis (95% CI = 1.65–2.22, 95% CI = 3.37–4.45, 95% CI = 7.64–1.14, and 95% CI = 8.66–13.13, respectively). Interestingly, it was found that the odds of the blood donors to be detected with syphils based on their age was increasing with increase in their age (Table 2). Blood donors who were residing in Kilimanjaro were protected from from contracting the syphilitc infection (COR = 0.91). On the other hand, those who were residing in Manyara, Tanga, and other regions were 1.49 and 1.26 times more likely to be detected with syphilis than those who were residing in Arusha. Table 2 presents the association between the different TTIs and age, sex, and type of blood donors.

## Discussion

Our study has highlighted that overall prevalence of TTIs in the donated blood in Northern Tanzania was 10.1%. Most of the blood donors (98%) were first time donors while voluntary non-remunerated blood donors accounted for the most (78.3%) of the donors. Considering the socio-demographic characteristics of the study subjects, the majority of participants were males, with a male to female ratio of 5.1:1. High proportion of male blood donors over females is not new. Previous studies have indicated very high male to female ratio [14, 17]. The possible explanation for this observation could be due to the high deferral rates of potential female donors because of anemia, pregnancy, breastfeeding or childbirth which are all criteria for donor exclusion. Likewise, the observation could be attributed to the cultural myth that women should abstain from blood donation as they regularly lose blood via menstruation.

Most (44.7%) of the blood donors in our study were young aged between 18–25 years. These findings are similar with a study in Kenya where 59% of voluntary donors were <25 years old [18]. Our study participants’ mean ages are 10–15 years less than those observed in western countries [19]. The possible explanation for this difference could be explained by the fact that the voluntary donor programs in Africa tend to be centered on secondary school and university students [20].

The present study has found that majority (78.3%) of blood donors were voluntary non-remunerated donors with remaining proportion being replacement donors. The findings compare well with a previous study which indicated that voluntary non-remunerated blood donors were nearly 90% of all blood donors [14]. On the contrary, another study found a reverse composition where replacement donors were more than voluntary non-remunerated blood donor [21]. The reason for this difference is not clear; however, this might be due to differences in time and geographical locations where these studies have been done, and/or awareness towards blood donor eligibility among populations where these studies were conducted. The voluntary non remunerated blood donors are generally considered safer than replacement donor. WHO and other blood donor organizations recommend that all blood donation should be voluntary and non-remunerated and that no coercion should be brought to bear upon the donor to donate [13, 22].

In this study we found that males were 2 times more likely to be infected with HBV than females. HBV infection often occurs during infancy and childhood by horizontal transmission among children and young adults. The higher prevalence rate of HBV among relatively older people in the current study may suggest that most of these participants may have been infected at earlier stage of their life. Alternatively, they may have contracted the infection through sexual contacts [20]. The sub-Saharan region is highly endemic with HBsAg carrier rates of 9–20%, whereas 56–98% of the adult population shows evidence of past exposure to HBV infection [23–26].

Tanzania like many other sub-Saharan African countries continues to face critical challenges in blood availability and safety. The high prevalence of blood borne viruses and other infectious diseases, including HIV, HBV, HCV and syphilis remain to be a major concern. The risk of TTIs is still an important issue in Tanzania despite the recent published reports suggesting that significant progress has been made in countries where WHO national haemo-vigilance protocols have been implemented [22]. Therefore, awareness of this information is important in monitoring the magnitude and trends of a spectrum of TTIs in donor population for the effective intervention strategies.

The present study has highlighted that overall prevalence of TTIs in the donated blood was at 10.1% and HBV was the most prevalent TTI followed by HIV. Previous studies have reported similar trend [14, 25]. However, the observed prevalence in the current study is much higher than the findings from a study done in Eritrea which reported the prevalence of 3.7% [17]; and lower than prevalence reported by a Nigerian study which reported the prevalence of 28.8% [26]. The difference in the prevalence is not well clear; however, it could be due to differences in health care systems in the different study settings as well as varying magnitude of risk factors for contracting TTIs in the various settings.

The overall high prevalence of the cumulative frequency of TTIs found in our study is in line with the results from a previous study which highlighted that TTIs contributed nearly to two thirds (62%) of all deferred blood that was donated [14]. TTIs are well known among the commonest causes of blood donor deferrals determined by the positivity of blood screening tests [14]. TTIs that are routinely screened in the Tanzanian Northern Zone Blood Transfusion Center (NZBTC) include HBV, HCV, HIV, and Syphilis. The high prevalence of TTIs observed in the present study agrees well to the nature of low-and middle-income countries where there is a double burden of infectious diseases and non-communicable diseases including cancers [23]. The burden of infectious and communicable disease plus malnutrition is higher when compared to the high income countries [23].

Our study has found that the prevalence of donors with TTIs was higher (12.3%) among the replacement donors compared to the voluntary non-remunerated (9.5%), though the difference was not statistically significant. Similar observations have been reported elsewhere [9, 10], The findings suggest that family replacement donor blood is more likely to transmit TTIs than would voluntary donors possibly due to a number of factors including high risk behaviors and paid donors posing as close family members or relatives. The findings strongly indicate that replacement donors are less suitable and that major emphasis should be made to encourage voluntary non-remunerated blood donors.

The present study has highlighted that HBV is more prevalent among blood donors (5.1%) than HIV (1.9%). The prevalence of HBV in our study population can be explained by the high prevalence of this infection in the general population [25]. Individuals who are infected with HBV and HIV are permanently disqualified for blood donation and thus reducing the pool of potential donors in the general population. The overall prevalence of HIV in the current study was nearly equal among male and female donors. This is contrary to what was reported in studies done in Kenya [9] and in Nigeria [24, 27] which reported overall prevalence of HIV to be more in male than female. The prevalence of HIV among adults’ ages 15 to 64 years in Tanzania is estimated to be 7% (8% among females and 6% among males). This corresponds to approximately 1.7 million people living with HIV ages 15 to 64 years in Tanzania [26, 28]. Women of all ages are more likely than men to become infected with HIV during unsafe sex which may be attributed to cultural, socio-economic, and biological factors which have shown to contribute to the female genders’ vulnerability to HIV. The reason behind the nearly equal prevalence of HIV among male and female donors observed in this study is not clear. However, the high ratio of male to female study subjects in the present study may partly explain this observed difference.

Our study has found that blood donors residing in Tanga region were more likely to be detected with HIV-infection than other regions and the difference was statistically signifant. This can partly be explained by a relatively high prevalence of HIV in this region as compared to the others [28, 29]. We further noted that males were 1.19 times more likely to be detected with syphilis than females and the differnce was signficant. The findings compare well to the previous Kenyan study which have reported male dominance in syphilis positivity [9].

This study has a number of limitations. Firstly, the nature of study design, being a cross-sectional study, it does not establish which proceeded the other; whether TTIs or the risk factors. Secondly, being retrospective in nature, the present study was unable to control, or provides exhaustive information on risk factors for the TTIs which may have influenced our results. For instance, the range of risk factors evaluated in this study was limited. Importantly, information about the causes for deferrals in this study was lacking. Thirdly, a considerable proportion (5.5%) of the study subjects were excluded because of incomplete data thus a cautious interpretation of the study findings is warranted. Lastly but not least, seroprevalence determined by the kits used in this study may not necessarily amount to current infection.

## Conclusions

This study has shown at least 10% of the donated blood in the Tanzania Northern Zone Blood Transfusion Center harbors TTIs of which HBV accounted for about 5.2%. The prevalence of TTIs for replacement donors was relatively higher (12.3%) than that of voluntary non-remunerated donors (9.5%). Therefore, TTIs remain to be the greatest threats to blood safety in this setting thus strict adherence to selection and retention criteria including voluntary, non-remunerated low-risk blood donors are recommended to improve blood safety in the region. Also, strict adherence to algorithm of donor screening is highly recommended.

## Supporting information

S1 Dataset (RAR)
